# Obsessive-compulsive symptoms and related risk and protective factors in Black individuals in Canada

**DOI:** 10.3389/fpsyg.2025.1422900

**Published:** 2025-03-06

**Authors:** Elisabeth Dromer, Grace Jacob, Monnica T. Williams, Seyed Mohammad Mahdi Moshirian Farahi, Wina Darius, Cary Samuel Kogan, Jude Mary Cénat

**Affiliations:** ^1^Interdisciplinary Centre for Black Health, Faculty of Social Sciences, University of Ottawa, Ottawa, ON, Canada; ^2^School of Psychology, Faculty of Social Sciences, University of Ottawa, Ottawa, ON, Canada; ^3^Department of Psychology, Faculty of Arts and Social Sciences, Carleton University, Ottawa, ON, Canada

**Keywords:** obsessive-compulsive disorder, everyday racial discrimination, internalized racism, racial microaggressions, social support, Black individuals in Canada

## Abstract

**Background:**

Data from the United States showed that Black individuals face unique issues related to obsessive-compulsive disorder (OCD). However, Canadian research on OCD among Black individuals remains very limited. The present study aims to document obsessive-compulsive (OC) symptoms and related risk and protective factors in Black individuals aged 15 to 40 years old in Canada.

**Methods:**

A total of 860 Black individuals (75.6% female) aged 15–40 years were recruited as part of the Black Community Mental Health in Canada (BcoMHealth) project. Independent t-tests, ANOVA, and multivariable linear regressions were used to assess OC symptom severity and identify risk and protective factors.

**Results:**

Black individuals presented high levels of OC symptoms. Results showed that Black individuals born in Canada experienced more OC symptoms compared to those born abroad. Results also showed that there were no differences between Black women, Black men, and those who identified their sex as “other.” Everyday discrimination, internalized racism, and microaggressions positively predicted OC symptoms, while social support negatively predicted OC symptoms.

**Limitations:**

Limitations of this study include its cross-sectional nature, which prevents us from establishing causal links, not assessing for the clinical diagnosis of OCD, and using self-report measures. Results support that different forms of racial discrimination contribute to the development and severity of OC symptoms in Black individuals in Canada. Social support may play a protective role for those individuals. These factors must be considered in future research and in the assessment and treatment of Black individuals with OCD.

## Introduction

Obsessive-compulsive disorder (OCD) is a potentially debilitating condition that impacts the individuals who suffer from it as well as those close to them. OCD can vary in its presentation. In fact, people from differing ethnic groups appear to vary in their symptomology. In particular, Black individuals in the United States (US) may be more likely to engage, among others, in double checking (a common OCD symptom), which has been attributed to this group’s unique experience of anti-Black racism both presently and historically (Williams et al., 2017c). However, little is known of this disorder in Canada, especially in Black individuals. There is a need to address this knowledge gap in order to better understand the unique aspects of their experiences, so as to better tailor treatment to this group.

OCD is a disorder characterized by the presence of time-consuming and distressing obsessions (unwanted and recurrent thoughts) and/or compulsions (repetitive behaviors or mental acts used to manage obsessions) (American Psychiatric Association, 2013; World Health Organization, 2019). Worldwide, its prevalence is estimated to be 1.5% in women and 1.0% in men (Fawcett et al., 2020). Research on its prevalence in Canada is scarce. Data from the *2012 Canadian Community Health Survey—Mental Health* showed the lifetime prevalence of OCD to be 0.93% (95% CI 0.75–1.11) for Canadians over the age of 15 (Osland et al., 2018). Additionally, Canadian prevalence data on OCD is limited in that it does not disaggregate by race or ethnicity—a color-blind approach very common in Canadian health research (Fante-Coleman and Jackson-Best, 2020; Fryberg and Stephens, 2010).

### OCD in Black individuals in a minority context

The majority of research on OCD has been conducted in populations from the US. However, until less than two decades ago, these studies did not investigate OCD within Black populations (Williams et al., 2010). The first comprehensive study of OCD among African Americans and Black Caribbeans found 12-month and lifetime rates of OCD among them of 1.63 and 1.49% respectively, suggesting similar prevalence rates to the general population (Himle et al., 2008). There is also a dearth of data on risk and protective factors for OCD in Black individuals. Sociocultural risk factors for OCD in African American children and youth identified, to date, include low income and perceived racism, while identified protective factors include items such as social support and religious coping (Williams and Jahn, 2016). Other studies by Williams and colleagues also found that everyday racial discrimination (but not non-racial discrimination) was positively associated with OCD obsessions (Williams et al., 2021; Williams et al., 2017c). Hence, while research on OCD in Black populations is limited, the current data show a prevalence in Black communities similar to that found in the general population and suggest important links between OCD and racial discrimination.

### Association between racial discrimination and OCD

The connection between racial discrimination and OC symptoms was revealed by multiple studies conducted in the US by Williams and other researchers during the last decade (Williams and Jahn, 2016; Williams et al., 2017c; Wilson and Thayer, 2020). These studies revealed that Black individuals who experience racial discrimination are more likely to experience OC symptoms. Indeed, it is well known that experiencing racial discrimination is related to an increase in stress, which in turn leads to deleterious effects on the health of individuals (Cénat, 2022). Concretely, higher levels of experienced racial discrimination result in a worsening of many pre-existing mental health conditions, such as OCD (Lewis and Neville, 2015; Williams et al., 2017c). However, beyond a general stress process model, unwanted obsessions and unwanted compulsions can also be a function of the nature of racism. For example, anti-Black racism can take the form of assumptions of intellectual inferiority, which may be operationalized as excessive criticism for minor mistakes towards Black individuals. In turn, to prevent negative judgment, Black individuals may engage in excessive double-checking, a common OCD compulsion (Williams et al., 2017c). Thus, racial discrimination may be indirectly associated with OCD because of its inherent stressful nature, and it may also directly impact OCD and the nature of Black individuals’ obsessions and compulsions.

Very rarely will people who enact racism admit they are acting out of racial animus. Hence, when people experience racism, it is common for targets to believe they are being mistreated due to negative qualities about themselves (Williams et al., 2020). Therefore, lacking an explanation for being treated worse than others, many Black people start to believe they are defective in some way and may adopt negative stereotypes about their racial group. This is called *internalized racism* and is connected to numerous negative mental health outcomes (Cénat et al., 2022; Haeny et al., 2021; Kogan et al., 2022). Internalized racism has never been studied in connection with OCD, but one might predict that, like other forms of racism, it is a risk factor for OCD.

### Purpose of study

Although symptoms of OCD in Black Canadians may be comparable the general population, there are distinct factors that affect OCD among Black people. Thus, this study aims to inform our understanding of OCD in Black Canadians by: (1) assessing their self-reported obsessive-compulsive (OC) symptoms and (2) examining possible risk and protective factors associated with OC symptoms in this population.

## Methods

### Participants

A total of 860 Black individuals (75.6% female) aged 15–40 years (*M* = 24.96 years, SD = 6.29) and living in Canada were recruited as part of the Black Community Mental Health in Canada (BcoMHealth) project. Descriptive statistics are reported in Table 1. Details on the recruitment methods used in this study and more in-depth descriptions of the sample can be found in previous publications (Cénat et al., 2021a; Cénat et al., 2021b). Upon giving consent, participants completed the online survey on Qualtrics™ (Provo, USA), where they filled out a number of questionnaires, including the ones presented in this study. Respondents received compensation either through a course credit, or a $15 e-gift card, depending on the situation. Ethical approval was obtained from the University of Ottawa and Université du Québec en Outaouais research ethics boards.

**Table 1:** Sample characteristics and OC symptoms.

	Total (*N* = 860) Percent	OC symptoms, *M* (*SD*)	Significance test ^1^
Age, M (SD)	24.96 (6.29)		6.30 (781.23), <0.001
<24 years old	64.77	12.87 (6.75)	
> 25 years old	35.23	15.43 (5.00)	
Sex			1.88 (2, 857), 0.153
Male	21.05	12.97 (7.05)	
Female	75.58	13.98 (6.01)	
Other	3.37	14.07 (7.65)	
Employment			2.23 (538.41), 0.026
Employed	65.93	14.13 (6.05)	
Unemployed	34.07	13.09 (6.73)	
Education			9.05 (4, 855), <0.001
No high school diploma or certificate	5.23	10.36 (7.26)	
High school diploma or equivalency certificate	14.53	12.78 (7.61)	
No post-secondary degree, certificate or diploma but received some post-secondary education	56.05	14.72 (5.18)	
Post-secondary certificate or diploma below bachelor level	16.74	13.49 (6.95)	
Bachelor’s degree, university certificate or diploma above bachelor level	7.44	11.63 (7.38)	
Place of birth			6.59 (241.54), <0.001
Canada	79.07	14.58 (5.76)	
Abroad	20.93	10.72 (7.28)	
Marital status			18.72 (3, 856), <0.001
Single	51.63	12.27 (7.14)	
Married	38.72	15.46 (4.26)	
Separated	5.93	14.55 (5.75)	
Other	3.72	15.75 (7.56)	
Religion			15.23 (5, 854), <0.001
Christian	33.14	11.48 (7.08)	
Hindu	2.67	15.74 (7.05)	
Muslim	42.56	15.38 (4.82)	
Buddhist	2.91	16.00 (6.56)	
Other	6.98	14.77 (7.17)	
None	11.74	12.83 (5.90)	

1 t (df), *p*-value and F (df_1_, df_2_), *p*-value for variables with two categories and variables with more than two categories were reported, respectively.

### Measures

#### Sociodemographic information

This questionnaire was used to assess sociodemographic information (e.g., gender, education level, marital status, place of birth, religion, etc.).

#### Everyday racial discrimination scale (EDS)

This questionnaire consists of 5 items assessing the perceived sources of racial discrimination that individuals face and the extent to which they are present in the respondent’s life (Williams et al., 1997). For example, one item is “because of your skin colour: you are treated with less courtesy.” This scale is rated on a 6-point scale ranging from “*Almost every day”* to “*Never.”* A high score indicates that there is a high incidence of perceived racial discrimination. Cronbach’s alpha: 0.90.

#### Internalized racism scale

This questionnaire contains 12 items that assess the extent to which an individual agrees with certain stereotypical statements related to their ethnic group and race (e.g., straight hair is better than my natural hair texture) (Molina and James, 2016). This scale is scored on a 4-point scale ranging from “*Very true” to* “*Not true at all.”* It has no formal scoring, but a high score indicates a greater prevalence of internalized racism. The following items must be reverse scored: 1 and 3. The Cronbach’s alpha of our sample was 0.78.

#### Inventory of microaggressions against Black individuals (IMABI)

This questionnaire is composed of 14 items assessing the experiences of racial microaggressions (i.e., microinsults, microassaults) among Black individuals (Mercer et al., 2011). For example, one item includes “Someone told me that I am not like other people of my racial/ethnic background.” This scale is rated on a 5-point scale ranging from “*This has never happened to me”* to “*This event happened to me and I was extremely upset*.” A high score indicates that the incidence of racial microaggressions is high. Cronbach’s alpha in the present sample was 0.86.

#### Multidimensional scale of perceived social support

This questionnaire consists of 12 items evaluating the sources of social support of an individual, which are divided into three groups: friends (items 6, 7, 9, and 12), significant other (items 1, 2, 5, and 10) and family (items 3, 4, 8, and 11) (Zimet et al., 1988). An example item is “I can count on my friends when things go wrong.” This scale is scored on a 6-point scale from “*Very Strongly Disagree,”* to “*Very Strongly Agree.”* The Cronbach’s alpha coefficients were of 0.85 for friends, 0.91 for significant other, and 0.87 for family (total reliability score of 0.88) (Zimet et al., 1988). The Cronbach’s alpha in our sample was 0.90.

#### Yale-Brown obsessive-compulsive scale (Y-BOCS)

This questionnaire contains 10 self-rated items assessing the severity of obsessions and compulsions (Goodman et al., 1989). For example, one item is “How much of your time is occupied by obsessive thoughts.” This scale is rated on a 5-point scale, and ranges from 0 (no symptoms) to 4 (severe symptoms), and participants can receive a score between 0 and 40. A higher score represents more severe symptoms of obsessions and compulsions (Goodman et al., 1989; Steketee et al., 1996). More precisely a total score between 0 and 7 indicates subclinical levels of symptoms, 8–15 mild symptoms, 16–23 moderate symptoms, 24–31 severe symptoms, 32–40 extreme symptoms (Wootton and Tolin, 2016). The Cronbach’s alpha of our sample was 0.87.

### Statistical analyses

To compare means of OC symptoms between the sociodemographic characteristics of the sample, we used independent t-tests (for the variables with two categories) and one-way ANOVAs (for the variables with more than two categories). A series of pairwise comparisons using Tukey’s test were performed. Bivariate correlations between continuous variables were tested using Pearson’s *r* correlations (Table 2). Following this, hierarchical multiple linear regressions were conducted with three models to see what variables significantly predicted OC symptoms. Model 1 included sociodemographic variables. In Model 2, variables related to the experience of racial discrimination were added. In Model 3, social support was included. Standardized scores of the continuous variables were used for the regression analysis. Categorical variables included age, sex, education, marital status, employment status, place of birth, and religion. Age was also included in the regression analysis as a continuous variable. The multicollinearity assumption was checked using the variance inflation factor (VIF). We only included sociodemographic variables which were significant in the preliminary multiple regression analysis (see Supplementary Table S1 for other variables). All statistical analyses were performed using SPSS 28 and Stata/SE 16.1.

**Table 2:** Zero-order correlations.

	1	2	3	4	5	6
1. OCD symptoms	1					
2. Everyday discrimination	0.352**	1				
3. Internalized racism	0.444**	0.416**	1			
4. Microaggression	0.257**	0.348**	0.152**	1		
5. Social support	−0.295**	−0.220**	−0.324**	−0.078*	1	
6. Age	0.169**	0.362**	0.225**	−0.030	−0.091*	1

*<0.05, **<0.001.

## Results

ANOVAs and t-test results are displayed in Table 1. Participants reported, on average, mild levels of OC symptoms (Total Y-BOCS score: *M* = 13.77, *SD* = 6.31). Results of the *t*-tests showed that mean OC score was significantly higher in participants who were born in Canada (*M* = 14.58, *SD* = 5.76) comparatively to those who were born abroad (*M* = 10.72, *SD* = 7.28), *t* (241.54) = 6.59, *p* < 0.001. Similar results were found for those who were employed (*M* = 14.13, *SD* = 6.05) compared to those who were unemployed (*M* = 13.09, *SD* = 6.73), *t* (538.41) = 2.23, *p* = 0.026. In addition, results showed that those aged 25 years old and more (*M* = 15.43, *SD* = 5.00) were more likely to experience OC symptoms compared to those aged 24 years old and less (*M* = 12.87, *SD* = 6.75), *t* (781.23) = 6.30, *p* < 0.001.

ANOVAs demonstrated significant differences among the different marital status (*F* (3, 856) = 18.72, *p* < 0.001), religions (*F* (5, 854) = 15.23, *p* < 0.001), and education level (*F* (4. 855) = 9.05, *p* < 0.001) of participants. Pairwise comparisons showed that married participants (*M* = 15.46, *SD* = 4.26) had a higher mean OC symptom score than single participants (*M* = 12.27, *SD* = 7.14). Moreover, single participants (*M* = 12.27, *SD* = 7.14) had a lower mean OC symptom score than participants who had “other” marital status (*M* = 15.75, *SD* = 7.56). Christians (*M* = 11.48, *SD* = 7.08) had lower mean OC symptoms compared to Muslims (*M* = 15.38, *SD* = 4.82), Buddhist (*M* = 16.00, *SD* = 6.56), Hindu (*M* = 15.74, *SD* = 7.05), and “other” religions (M = 14.77, SD = 7.17), *p*_Christian-Muslim_ < 0.001; *p*_Christian-Buddhist_ = 0.005; *p*_Christian-Hindu_ = 0.016; p _Christian-Other_ = 0.002. Furthermore, Muslims (*M* = 15.38, *SD* = 4.82) had a significantly higher mean OC symptom score than participants with no religion (*M* = 12.83, *SD* = 5.90). Results also showed that participants with no high school education (*M* = 10.36, *SD* = 7.26) had a lower mean OC symptom score than participants with an incomplete post-secondary degree (*M* = 14.72, *SD* = 5.18; *p* < 0.001) and those with a post-secondary degree (*M* = 13.49, *SD* = 5.95; *p* = 0.026). Participants with a high school degree (*M* = 12.78, *SD* = 7.61) had a lower mean OC symptom score than participants with an incomplete post-secondary degree (*M* = 14.72, *SD* = 5.18; *p* = 0.016). Participants with an incomplete post-secondary degree (*M* = 14.72, *SD* = 5.18) had a higher mean OC symptom score than participants with a bachelor’s degree/university certificate (*M* = 11.63, *SD* = 7.38; *p* = 0.002). No mean differences between sex groups were found, *F* (2, 857) = 1.88, *p* = 0.153.

Predictors of Model 1 (see Table 3) accounted for 8.7% of the variance. After adding everyday racial discrimination, internalized racism, and racial microaggressions to the model, *R*^2^ increased by 16.7% to a total of 25.4% of variance explained, suggesting a notable contribution of these three predictors to the model. After adding social support to the model, the *R*^2^ increased by 2.0%. In total, the predictors in the final model accounted for 27.4% of the variance. The results of the final model showed that experience of everyday discrimination (*B* = 0.63, *p* = 0.006), degree of internalized racism (*B* = 1.76, *p* < 0.001), and racial microaggressions (*B* = 1.05, *p* < 0.001) were positively associated with OC symptoms. Results also revealed that increased social support scores were negatively associated with OC symptoms (*B* = −0.96, *p* < 0.001). Age as a continuous variable was not a significant predictor of OC symptoms, despite the group differences observed when grouping participants in the two age categories (24 and under, and 25 and over).

**Table 3:** Hierarchical Multiple Regressions Results for OC Symptoms and Various Sociodemographic Variables.

	Model 1*R*^2^ = 0.087, *F* (3, 856) = 28.35, *p* < 0.001		Model 2*R*^2^ = 0.254, *F* (6, 853) = 49.70, *p* < 0.001		Model 3*R*^2^ = 0.274, *F* (7, 852) = 47.22, *p* < 0.001	
	Adjusted B (SE)^1^	*p-value*	Adjusted B (SE)^1^	*p-value*	Adjusted B (SE)^1^	*p-value*
Age	0.39 (0.23)	0.097	0.10 (0.22)	0.662	0.12 (0.22)	0.588
Place of birth	−3.25 (0.52)	<0.001	−0.97 (0.51)	0.057	−1.05 (0.50)	0.037
Marital status	1.14 (0.30)	<0.001	0.56 (0.28)	0.043	0.52 (0.28)	0.061
Everyday discrimination			0.74 (0.23)	0.001	0.63 (0.23)	0.006
Internalized racism			2.04 (0.22)	<0.001	1.76 (0.22)	<0.001
Microaggression			1.05 (0.20)	<0.001	1.05 (0.20)	<0.001
Social support					−0.96 (0.20)	<0.001

## Discussion

As the first Canadian study to explore OCD in Black communities in Canada, the present work provides new insights on risk and protective factors of OC symptoms in Black individuals.

### OC symptoms and demographic characteristics of the sample

There were some notable differences in symptom severity between various social groups. First, age was positively correlated with OC symptoms. Indeed, when categorized into two groups, individuals 25 years old and older had higher mean OC symptom scores than those 24-year-old and younger. These results make sense since participants in the sample are young. Indeed, in the US, Black individuals have a later identified age of onset for OCD (Himle et al., 2008), which could be due to barriers to diagnosis and treatment (Williams et al., 2012). Another reason discussed by Williams et al. (2017a,b,c) is that there are greater mortality rates among individuals with OCD than for those without the disorder. In addition, a study in Denmark found that individuals with OCD have a significantly higher mortality rate from natural and unnatural causes (Meier et al., 2016). Finally, being a part of a marginalized ethnic group could have an impact and increased the rate of OC symptoms among 25 years old and older Black individuals due to barriers accessing early interventions and effective treatment (Williams et al., 2017b).

OC symptoms were higher among Canadian-born individuals compared to those coming from abroad. In Canada, one in four Black person is born in the country (Domey and Patsiurko, 2024). Research has shown that when immigrants arrive to Canada, they are in better health than Canadian-born individuals (Beiser, 2005). This is known as the healthy immigrant effect. However, their health declines as they spend more time in their new country (Elshahat et al., 2022; Ng, 2011). Selection effects can also have an impact on the selection of immigrants who are healthier into Canada. Indeed, health screenings by host countries ensure that the healthiest citizens are selected for immigration (Kennedy et al., 2015), leaving “unhealthy” migrants in their country unable to immigrate. These phenomena could explain the presence of lower OC symptoms found in this study among Black individuals not born in Canada.

OC symptoms were higher among certain religious groups. Notably, Muslims had higher symptoms than Christians. This may be due to the cultural practices of the religions. As described in Yorulmaz et al. (2009), there are predetermined behavioural requirements in Islam, as this is a ritualistic religion. There rituals place an emphasis on purity, cleanliness, and prayers. These features of the religion could contribute to presence of OC symptoms and, when done to the extreme, can resemble OCD (Yorulmaz et al., 2010).

Finally, individuals with some post-secondary education and those with a post-secondary certificate had more OC symptoms compared to individuals with less or more education. While previous research has highlighted the negative impact of OCD on educational attainment in Black individuals (Himle et al., 2012; Williams et al., 2017b)—wherein individuals with OCD are less likely to receive a high level of education because of the impact of their symptoms—the contrary is not a common finding of the literature on OCD. Indeed, the sample in this study showed that those with an incomplete high school diploma had the lowest OC symptom score. This finding could be explained by the low age of our sample: many individuals may have simply not completed high school *yet*. In addition, individuals who have not completed high school represent only a small percentage of the sample in this study. Hence, results from such a small sample size should be interpreted with caution.

### OCD and experiences of racial discrimination

This study shows that everyday racial discrimination and microaggressions are predictors of obsessive-compulsive symptoms, which is consistent with US work reporting a significant association between OCD symptoms and racism (Williams et al., 2017c). Studies have hypothesized that the psychological resources necessary to deal with experiences of racial discrimination can diminish the resources available to manage other stressors, in this case, obsessions and/or compulsions. This, in turn, may lead to an increase in OC symptoms (Soto et al., 2011; Williams et al., 2017c). In addition, distress caused by racial discrimination was found to be a bigger predictor of OC symptoms than the frequency of this discrimination. Everyday racial discrimination is associated with serious psychological distress among African Americans (Chae et al., 2011). Hence, the distress associated with experiences of racial discrimination could also deplete the psychological resources that are available for Black individuals, which in turn could also lead to increased OC symptoms. Experiences of racial discrimination thus impact OCD in two ways that both lead to depleted resources and ultimately increased OC symptoms: individuals have to deal directly with the experiences of racial discrimination and have to deal with the psychological distress caused by these experiences.

### OCD and internalized racism

Interestingly, internalized racism was the largest predictor of OC symptoms in Black Canadians in this study. This is the first time this construct has been measured in the context of OC symptoms, making this finding particularly notable. Internalized racism is linked to an array of negative mental health outcomes, such as increased anxiety and shame (Graham et al., 2016; Speight, 2007). Both have also been associated with OCD (Stein et al., 2010; Szentágotai-Tătar et al., 2020; Visvalingam et al., 2022). Hence, individuals with higher levels of internalized racism may experience more shame and/or anxiety. This could lead to more negative self-talk and more anxious thoughts, which in turn could lead to increased OC symptoms, and explain the relationship between internalized racism and OCD. More research on the link between OCD and internalized racism is needed.

### OCD and social support

Williams and Jahn (2016) theorized that social support was a protective factor for Black people in terms of the development of OCD. Correspondingly, we found that social support was negatively correlated with OC symptoms in our sample. Previous research on OCD and its relation to family relationships in Black Americans has highlighted the negative impact of OCD on family relationships and the impact of negative family interactions on family relationships (Himle et al., 2017). Hence, we theorize that individuals who report high levels of social support are experiencing the opposite phenomenon, wherein experiencing positive social interactions and support helps them deal with obsessions and compulsions in a way that reduces OC symptoms. For example, if instead of being criticized for their compulsions, individuals are met with kindness and understanding, they may experience fewer of them. This theory has been highlighted in previous work on OCD and social support in the general population (Palardy et al., 2018).

### Limitations and future research

Some limitations should be considered. First, due to the cross-sectional nature of the study, causal links between OC symptoms and other variables, such as internalized racism, cannot be established. In addition, not assessing for the clinical diagnosis of OCD directly prevents us from establishing the prevalence of OCD in Black communities in Canada—which has yet to be done and is needed. Similarly, not collecting information concerning the nature of the obsessions and compulsions also limits our ability to paint a full portrait of OCD in Black individuals in Canada. Not assessing the participants’ use of psychopharmacological treatments also limits the generalizability of our findings. It is information that could be asked to be sure that those who declared fewer symptoms of OCD do not receive medication to manage them. Finally, using self-report measures may have limited the accuracy of the information obtained.

The results of this research help to highlight future avenues for research. First, future studies should investigate the prevalence of OCD symptoms and diagnoses in Black individuals in Canada. These studies should also explore the differences between Black Canadians with OCD and their White and other racialized counterparts. Studies should specifically investigate the experiences related to the types of symptoms (obsessions and compulsions) and how they impact the daily life of Black Canadians compared to other racial and ethnic groups. Second, future studies with longitudinal design should continue to examine the role of different forms of racial discrimination as risk factors for OCD and related symptoms. Internalized racism—as one of the most pervasive and damaging consequences of racial microaggressions and individual, institutional, and systemic racism—should especially be included in such work. Longitudinal studies are needed to better understand the mechanisms underlying the links between internalized racism, other forms of racism, and OC symptoms to find ways to mitigate their impact on OCD. Third, further research is needed on protective factors related to OCD in Black individuals to understand the mechanisms through which these factors lead to lower levels of OC symptoms. Such research is crucial for both OCD prevention and intervention, and to build resilience in Black communities and other ethnic and racial groups.

## Conclusion

This study highlights a number of risk and protective factors of OC symptoms in Black individuals in Canada. These include place of birth, employment status, marital status, religious affiliation, and education. Additionally, everyday racial discrimination and internalized racism seem to be particularly important predictors of OC symptoms in Black people living in Canada. These findings should inform future research and treatment of OCD among Black people in Canada. They also highlight the importance of exploring the impact of racism, and more specifically everyday racial discrimination, on psychological symptoms, which is consistent with studies published on depression and anxiety (Cénat et al., 2021b; Kogan et al., 2022). Although cross-sectional, these findings should inform future studies on the evaluation and treatment of OCD among Black individuals in Canada. This is necessary so that assessment and treatment of OCD can be provided to Black individuals in a culturally appropriate and anti-racist manner (Cénat, 2020; Cénat et al., 2024).

## Data Availability

The raw data supporting the conclusions of this article will be made available by the authors, without undue reservation.
