# Supplementary material for: Obsessive-compulsive symptoms and related risk and protective factors in Black individuals in Canada
Source: Front Psychol. 2025 Mar 6;16:1422900. doi: 10.3389/fpsyg.2025.1422900 (PMC11924203; doi:10.3389/fpsyg.2025.1422900)
Supplement: Supplementary file 1 [file Table_1.docx]

Supplementary Material

# Supplementary Table

**Table S1.** Multiple regression: associations between sociodemographic variables and OCD scores

|  | *R*^2^ = .087, *F* (7, 852) = 12.64, *p* <.001 | |
| --- | --- | --- |
|  | **Adjusted B (SE) ^1^** | ***p-value*** |
| **Age** | 1.07 (.21) | <.001 |
| **Sex** | -.19 (.51) | .713 |
| **Education** | .10 (.24) | .662 |
| **Employment** | -.24 (.48) | .625 |
| **Place of birth** | -3.07 (.54) | <.001 |
| **Marital status** | 1.03 (.35) | .003 |
| **Religion** | .10 (.14) | .496 |

**
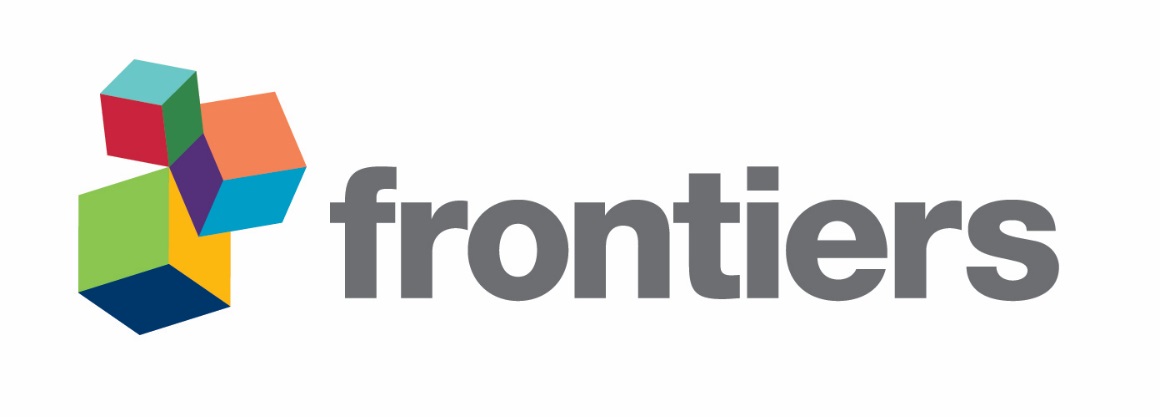
**
